# Impaired Executive Function and Depression as Independent Risk Factors for Reported Delirium Symptoms: An Observational Cohort Study Over 8 Years

**DOI:** 10.3389/fnagi.2021.676734

**Published:** 2021-06-07

**Authors:** Christian Mychajliw, Matthias L. Herrmann, Ulrike Suenkel, Katharina Brand, Anna-Katharina von Thaler, Isabel Wurster, Rezzak Yilmaz, Gerhard W. Eschweiler, Florian G. Metzger

**Affiliations:** ^1^Department of Psychiatry and Psychotherapy, University Hospital of Tübingen, Tübingen, Germany; ^2^Geriatric Center, University Hospital of Tübingen, Tübingen, Germany; ^3^Department of Neurology and Neuroscience, Medical Center-University of Freiburg, Faculty of Medicine, University of Freiburg, Freiburg, Germany; ^4^Department of Neurology, University Medical Center Schleswig-Holstein, Kiel, Germany; ^5^Department of Neurology, University Hospital of Tübingen, Tübingen, Germany; ^6^German Center of Neurodegenerative Diseases (DZNE), University of Tübingen, Tübingen, Germany; ^7^Department of Neurology, University of Ankara Medical School, Ankara, Turkey; ^8^Vitos Hospital for Psychiatry and Psychotherapy Haina, Haina, Germany

**Keywords:** delirium, executive function, depression, cohort study, observational study, acute encephalopathy, cognitive functions, cognition

## Abstract

**Background:**

Acute medical illnesses, surgical interventions, or admissions to hospital in older individuals are frequently associated with a delirium. In this cohort study, we investigated the impact of specific cognitive domains and depression before the occurrence of delirium symptoms in an 8-year observation of older non-hospitalized individuals.

**Methods:**

In total, we included 807 participants (48–83 years). Deficits in specific cognitive domains were measured using the CERAD test battery, and depressive symptoms were measured using Beck Depression Inventory and the Geriatric Depression Scale (GDS) before the onset of a delirium. Delirium symptoms were retrospectively assessed by a questionnaire based on the established Nursing Delirium Screening Scale.

**Results:**

Fifty-eight of eight hundred seven participants (7.2%) reported delirium symptoms over the 8-year course of the study. Sixty-nine percent (*n* = 40) of reported delirium symptoms were related to surgeries. In multivariate regression analysis, impaired executive function was an independent risk factor (*p* = 0.034) for the occurrence of delirium symptoms. Furthermore, age (*p* = 0.014), comorbidities [captured by the Charlson Comorbidity Index (CCI)] (*p* < 0.001), and depression (*p* = 0.012) were significantly associated with reported delirium symptoms.

**Conclusion:**

Especially prior to elective surgery or medical interventions, screening for impaired executive function and depression could be helpful to identify patients who are at risk to develop delirium symptoms.

## Introduction

Delirium is a common neuropsychiatric syndrome, defined by acute decline and fluctuation of attention, cognitive function, and disturbance of consciousness in DSM-5 (American Psychiatric Association, 2013). It is associated with cognitive impairment, dementia, higher rates of institutionalization, and increased morbidity and mortality (Witlox et al., 2010). Incidence of delirium in older subjects ranges from 11 to 51%, depending on the setting (Inouye et al., 2014). Especially in older subjects, several factors such as multimorbidity, polypharmacy, age, and deficits in sensory and mobility function can increase vulnerability to delirium. Numerous studies demonstrated that cognitive impairment is one of the leading risk factors for delirium in hospitalized patients (Fong et al., 2015). However, only few population studies investigated the association between cognitive function and delirium in healthy individuals (de Lange et al., 2013; Davis et al., 2014; Daiello et al., 2019). Therefore, little is known about the impact of specific cognitive domains on the occurrence of delirium symptoms. Many reported cases of delirium are related to surgery and anesthesia (Martins and Fernandes, 2012). Yet, a delirium can also occur without surgery or trauma but in relation with fever, starvation, medication, or several kinds of illnesses (Rockwood et al., 1999; Rahkonen et al., 2001; Berger et al., 2018; Wilson et al., 2020).

With increasing age, there is a growing risk for neurodegenerative diseases in general (Mayeux, 2003; Erkkinen et al., 2018). Mild and slow decrease in cognition is common as an age-related process but can also be caused by neurodegeneration (Lövdén et al., 2010; Park and Bischof, 2013). Dementia, for instance, is a major matter of expense in healthcare and nursing systems (Quentin et al., 2010; Leicht et al., 2011; Wimo et al., 2017) and causes enormous individual suffering. Several studies showed different interactions between delirium and cognitive decline (Jackson et al., 2004; Greene et al., 2009; Inouye et al., 2016). Some authors propose that a delirium can be followed by cognitive decline (Devore et al., 2017; Sauër et al., 2017), even in the long term (MacLullich et al., 2009; Gross et al., 2012; Saczynski et al., 2012). In this case, postoperative delirium (POD) and Postoperative Cognitive Dysfunction (POCD) can be seen as diverging concepts, yet they seem to have common risk factors and antecedents (Daiello et al., 2019). Other authors suggest that cognitive impairment can increase the risk of a delirium following surgery (Krogseth et al., 2016). Moreover, enhanced neuroinflammation is associated with higher age. Neuroinflammation in aging is described by the term inflammaging (Franceschi et al., 2018). It encompasses the processes of systemic inflammation through continuing physiological stimulation of the immune system during aging (Franceschi et al., 2018). On a clinical level, a vulnerability can manifest in multimorbidity and maladaptive sickness behavior (Cunningham and MacLullich, 2013). This can be measured, for example, by the concept of frailty (Abellan van Kan et al., 2008a, b; Woo et al., 2015). Taken together, vulnerable and multimorbid frail patients are more likely to develop delirium (Cunningham and MacLullich, 2013; Watt et al., 2013; Wilson et al., 2020).

While many studies only include short screening tests to assess cognitive impairment, few use more distinguished cognitive assessments (Jackson et al., 2004; MacLullich et al., 2009). Developing effective screenings, more differentiated reports on cognitive changes as risk factors and consequences of a delirium might be helpful. Thus, impaired executive functions have been shown to increase the risk of delirium after surgery (Rudolph et al., 2006). Also, depression was found to increase the risk of POCD and delirium (Tully et al., 2010; Oldham et al., 2019). Geriatric depression seems to be related with cognitive deficits in general (Brailean et al., 2017), especially in executive functions (Patience et al., 2019). Moreover, depression is known to be related to cognitive decline in Alzheimer’s disease (AD) (Butters et al., 2008; Byers and Yaffe, 2011) and Parkinson’s disease (PD) (Stefanova et al., 2006; Santangelo et al., 2009; Barone et al., 2011; de la Riva et al., 2014). Depressive symptoms can occur as a prodrome, as an accompanying symptom, or as a consequence (Bennett and Thomas, 2014; Rock et al., 2014; Snowden et al., 2015). Moreover, symptoms of depression, dementia, and delirium can overlap and appear to be similar (Schuurmans et al., 2001; Givens et al., 2009; Köhler et al., 2010; Downing et al., 2013; Arnold, 2014). These associations are explicable, among other things, by neurobiological mechanisms: neuroinflammation (Hegeman et al., 2015; Franceschi et al., 2018) as well as changes in neurotrophin signaling modulate both depression (Naismith et al., 2012; Villanueva, 2013) and delirium (Wilson et al., 2020). Research implies that there is a connection between deficits in executive function and depression, delirium, and POCD (Greene et al., 2009; Smith et al., 2009).

Screening for risk factors based on differentiated information on specific cognitive domains can help take precautions to prevent delirium and POCD (Tieges et al., 2020; Calf et al., 2021). More specific and efficient tasks can be chosen for pre-intervention assessments as they take less time. According to recent guidelines, identification of subjects at risk (Hempenius et al., 2015; Siddiqi et al., 2016) can be helpful for choosing patients for prophylaxis, surveillance, and interventions in connection with surgeries (Baron et al., 2015; Aldecoa et al., 2017; Berger et al., 2018; Burton et al., 2019; Hughes et al., 2020). Hence, the aim of this study was to investigate the association between deficits in specific cognitive domains and reported delirium symptoms in a long-term observation over 8 years. Based on previous studies (Greene et al., 2009; Smith et al., 2009), we hypothesized that impaired executive function is associated with a higher risk of delirium symptoms. Moreover, we expected further established risk factors like age, comorbidities, and frailty to be found predictive in the present analysis.

## Materials and Methods

As a longitudinal prospective follow-up study, the TREND study (Tübinger evaluation of Risk factors for the Early detection of NeuroDegeneration, www.trend-studie.de) was initiated in 2009 to explore risk and prodromal markers for AD and PD. The sample is composed of 1,201 older subjects (age: 48 years and older), without AD or PD at the time of study entry, but with and without probable risk factors for AD and PD: hyposmia, rapid eye movement sleep behavior disorder, and major depression (Berg, 2008, 2012).

Numerous neuropsychological, neurological, and anamnestic assessments were executed biennially. In the scope of neuropsychological testing, a German version of the CERAD-plus test battery (Morris et al., 1988, 1993; Aebi, 2002; Ehrensperger et al., 2010) was conducted. Based on described cognitive tests, domain scores for memory, language, executive, and visuospatial domain were calculated, according to Roberts et al. (2008). Briefly, memory domain comprises word list recall and figure recall, and language domain includes semantic and phonematic fluency as well as Boston Naming Test. Executive domain was defined by Trail Making Test Part B (TMT-B), and visuospatial domain was defined by figure copying (Petersen et al., 2009, 2010).

Moreover, symptoms of depression were examined with validated scales like Geriatric Depression Scale (GDS) (Sheikh and Yesavage, 1986; Gauggel and Birkner, 1999) and Beck Depression Inventory (BDI/BDI-II) (Beck et al., 1961, 1996a,1996b; Blaser et al., 1968; Kühner et al., 2007) at baseline. Nutritional state was assessed by the body mass index (BMI). Furthermore, comorbidities were evaluated by the expanded version of the Charlson Comorbidity Index (CCI) (Charlson et al., 1987), and physical status was measured using the FRAIL scale (Abellan van Kan et al., 2008a, b; Morley et al., 2012; Malmstrom et al., 2014). Since the comorbidities required for CCI as well as the FRAIL scale were not recorded at baseline examination, we used the mean value of the follow-up examinations.

To establish a method of evaluating delirium symptoms in TREND subjects, we developed a questionnaire based on the established Nu-DESC (Gaudreau et al., 2005; Lütz et al., 2008), as no validated questionnaire was available at the time of assessment. The Nu-DESC is a fast, simple, and systematic tool used for the diagnosis of a delirium (Gaudreau and Gagnon, 2005; Lütz et al., 2008), with a sensitivity of 86–96% and a specificity of 79–87% (Gaudreau and Gagnon, 2005; Gaudreau et al., 2005; Leung et al., 2008). As an alternative possibility to retrospectively assess delirium symptoms, Tsui et al. (2018) asked about delirium symptoms with one more general question (i.e., “Please think to a time when you have been unwell, perhaps while in hospital. Sometimes a person’s memory, thinking, and concentration can get worse over hours and days due to an illness, for example, infection, operation, or due to medications. This is delirium. Since 2006, have you experienced delirium symptoms?” Tsui et al., 2018). Another group conducted several interview questions with informants (Davis et al., 2014). In contrast to these examples, the questionnaire used in this study was designed to ask the subjects themselves on distinct delirium symptoms on a more nuanced level, i.e., symptom-wise. While the Nu-DESC is focused on current symptoms, the adapted questionnaire asked for delirium symptoms in the subjects’ individual medical history (“Have you experienced any of the following temporary symptoms at any time during your life in connection with a disease/injury described above?” cf. Supplementary Appendix). The given questionnaire was sent to all active subjects by mail. From the point of time of the conception of the questionnaire, it was established as a part of the routine study questionnaire given at each visit. After reviewing received questionnaires, ambiguous and incomplete answers were clarified by phone interview with the subjects, caregivers, and relatives (after given consent). Plausibility of cumulated answers was classified based on multi-professional expert consensus (experienced neurologist: MLH, geriatrician: GWE, and neuropsychologist: CM), as some described symptoms were more likely to be categorized as symptoms of different conditions such as dissociative states or panic attacks.

### Study Population/Selection of Subjects for Analysis

The questionnaire was sent to 993 participants in January and February 2018. A total of 920 questionnaires (92.6%) were returned within the following 6 months. Participants describing symptoms that are most likely not related to a delirium and could be better explained by another disease were excluded from the analysis (*n* = 31). Since the aim of this study was to evaluate predicting factors for delirium symptoms and previous deliriums could affect both memory and perception of future delirium symptoms, participants with a delirium prior to the study setup (prior to 2009) were also excluded (*n* = 47). Due to a possible bias of cognitive test results, we excluded all non-native speakers (*n* = 35) to avoid possible misinterpretation of cognitive testing. In total, 807 individuals were included in this analysis.

### Statistical Analyses

Differences between subjects with and without reported delirium symptoms were tested by χ^2^ test for categorical variables and by Mann–Whitney *U* test for non-normally distributed continuous variables. To analyze the association between specific cognitive domains and the occurrence of delirium symptoms, univariate logistic regression was performed for each domain. In a second step, the model was adjusted for covariates (age, sex, comorbidities, depression, frailty, and the sum of taken drugs). These are strong confounders based on previous studies and expert opinion. Receiving operating characteristic (ROC) curves with area under the curve (AUC) were calculated to predict participants’ individual risk of delirium symptoms over 8 years. Due to the study design, both the FRAIL scale and CCI were not assessed at each follow-up examination. Thus, we included means, respectively. To analyze the association between impaired executive function, delirium symptoms, and development of dementia over the 8-year observation period, we performed multivariate logistic regression. Data were analyzed with the software IBM SPSS Statistics Version 25 (IBM Corporation, Armonk, NY, United States). Results were considered statistically significant at a level of *p* < 0.05.

## Results

### Participants’ Characteristics

A total of 807 participants aged between 48 and 83 years were included in this study, the average age at baseline was 66.1 years. The baseline sociodemographic and clinical characteristics of all participants are described in Table 1. Participants reporting delirium symptoms were significantly older (*p* < 0.001), had more comorbidities (*p* < 0.001), and had higher scores in the FRAIL scale (*p* < 0.001) than participants without reported delirium symptoms. Furthermore, delirium patients were significantly more depressive (*p* < 0.001) at baseline assessment. No significant differences were found for sex (*p* = 0.314), years of education (*p* = 0.349), number of taken medicines (*p* = 0.578), and BMI (*p* = 0.885).

**TABLE 1:** Baseline characteristics for participants with and without delirium symptoms.

**Baseline characteristics**	**Participants without delirium symptoms**	***n***	**Participants with delirium symptoms**	***n***	***p*-value**
Age, in years, mean ± SD	62.9 ± 6.4	749	66.1 ± 6.6	58	**<0.001**
Female sex, *n* (%)	349 (46.6)	749	31 (53.4)	58	0.314
Years of education, mean ± SD	14.4 ± 2.6	749	14.9 ± 3.2	58	0.349
CCI, median (IQR)	1.0 (0.0–3.0)	744	3.0 (1.0–5.0)	58	**<0.001**
Number of drugs, median (IQR)	1.0 (0.0–2.0)	749	1.0 (0.0–3.0)	58	0.578
FRAIL scale, median (IQR)	0.0 (0.0–0.5)	720	0.5 (0.0–1.0)	57	**<0.001**
GDS, median (IQR)	1.0 (0.0–2.0)	745	3.0 (1.0–4.0)	57	**<0.001**
BDI-II, median (IQR)	5.0 (2.0–9.0)	744	11.0 (5.0–15.0)	56	**<0.001**
BMI in kg/m^2^, median (IQR)	25.1 (22.9–28.0)	749	25.3 (23.3–28.8)	58	0.885

*SD, standard deviation; IQR, interquartile range; CCI, Charlson Comorbidity Index; GDS, Geriatric Depression Scale; BDI, Beck Depression Inventory; BMI, body mass index. Significant p-values are marked in bold.*

### Delirium Symptoms

Of 807 participants, 58 (7.2%) reported delirium symptoms over the 8-year course of the study. In seven of these subjects, symptoms occurred in a period of 2 years after inclusion (12.1%), and in 11 participants (19.0%), between 2 and 4 years after enrollment. Another 15 subjects reported symptoms between 4 and 6 years (25.9%) after inclusion, and 24 subjects reported symptoms after 6–8 years (39.7%).

A large majority of delirium symptoms were observed after surgery (69.0%, *n* = 40) followed by other medical interventions such as colonoscopy or bronchoscopy (12.1%, *n* = 7). Six participants reported delirium symptoms because of inflammatory diseases (10.3%), and two more participants observed symptoms after other acute diseases (3.4%, pulmonary embolism and traumatic brain injury). In three cases, the exact trigger of delirium symptoms remains unknown.

### Association Between Cognitive Function, Depression, and Delirium Symptoms

As provided in Table 2, reported delirium symptoms are significantly associated with lower executive function (TMT-B, *p* < 0.001) as well as worse performance in word list recall (*p* = 0.016) and Boston Naming Test (*p* = 0.049) at baseline. No significant association was found for figure recall, semantic and phonematic fluency, as well as for the visuospatial domain (figure copying). After adjustment for age, comorbidities, frailty, and depressive symptoms in multivariate analysis, a significant association remained for the executive function (see Table 3).

**TABLE 2:** Cognitive characteristics from participants with and without delirium symptoms according to the CERAD-Plus test battery at baseline, *z* scores.

**Cognitive domain (*z* score at baseline)**	**Participants without delirium symptoms (mean ± SD)**	***N***	**Participants with delirium symptoms (mean ± SD)**	***n***	***p*-value**
**Global cognition**	−0.52 ± 1.01	749	−0.66 ± 1.03	58	0.376
MMSE	−0.52 ± 1.01	749	−0.66 ± 1.03	58	0.376
**Executive domain**	0.47 ± 1.12	745	−0.05 ± 1.05	58	**<0.001**
TMT-B	0.47 ± 1.12	745	−0.05 ± 1.05	58	**<0.001**
**Memory domain**	−0.17 ± 0.75	749	−0.23 ± 0.81	58	0.441
Word list recall	−0.07 ± 0.91	749	−0.34 ± 0.85	58	**0.016**
Figure recall	−0.27 ± 1.13	749	−0.12 ± 1.26	58	0.292
**Language domain**	0.31 ± 0.71	749	0.19 ± 0.76	58	0.120
Semantic fluency	0.16 ± 1.10	749	−0.17 ± 1.24	58	0.125
Phonematic fluency	0.43 ± 1.15	649	0.65 ± 1.15	46	0.251
Boston naming test	0.35 ± 0.75	749	0.12 ± 0.85	58	**0.049**
**Visuospatial domain**	−0.41 ± 1.15	749	−0.19 ± 1.11	58	0.248
Figure copying	−0.41 ± 1.15	749	−0.19 ± 1.11	58	0.248

*SD, standard deviation; MMSE, Mini-Mental State Examination; TMT-B, Trail Making Test B. Significant p-values are marked in bold.*

**TABLE 3:** Multivariate analysis of risk factors associated with delirium symptoms.

	**Odds ratio**	**95% Confidence interval**	***p-*value**
Age (in years)	1.060	1.012–1.110	**0.014**
Charlson Comorbidity Index	1.289	1.117–1.486	**<0.001**
FRAIL Scale	1.395	0.893–2.180	0.144
Geriatric depression scale	1.140	1.029–1.262	**0.012**
Executive function (TMT-B)	0.749	0.573–0.978	**0.034**
Word list recall	0.853	0.610–1.192	0.351
Boston Naming Test	0.733	0.520–1.034	0.077

*Cognitive parameters (TMT-B, Word list recall and Boston Naming Test) are presented as z values. Higher z scores indicate better cognitive performance. Significant p-values are marked in bold. Nagelkerke’s Pseudo-R^2^ = 0.205.*

**FIGURE 1:**
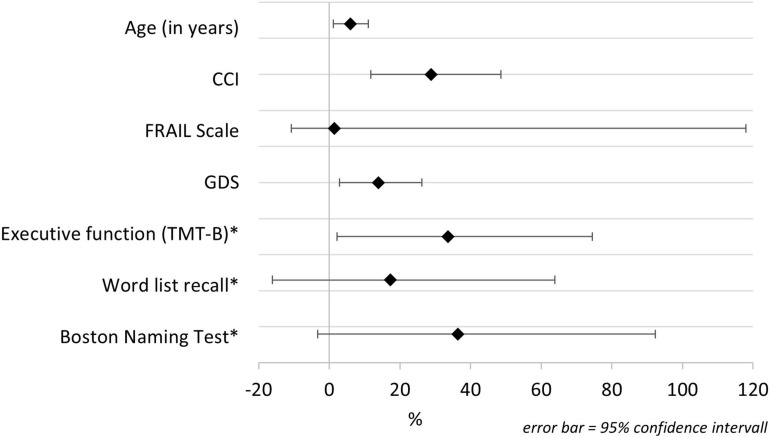
Change in the risk of reported delirium symptoms over an 8-year period. CCI, Charlson Comorbidity Index; GDS, Geriatric Depression Scale; TMT-B, Trail Making Test B. *Cognitive parameters are presented inverted.

The change in the risk of developing delirium symptoms over the 8-year period is shown in Figure 1. The change is illustrated as percentage value per additional unit of the analyzed parameter. Delirium risk increases by 6% per additional year of age and by 28.9% per additional disease (recorded with the CCI). The risk also significantly increases with additional depressive symptoms (14% for each point of GDS). Considering the cognitive parameters, only executive function has a significant influence on the risk of delirium symptoms over the 8-year period. Figure 1 demonstrates the change of risk in executive function, word list recall, and Boston Naming Test. Since the values of these parameters are given as inverted *z* scores, the changes of risk are related to one additional standard deviation of the *z* score. Each deterioration of the (*z* score adjusted) TMT-B by one standard deviation increases the probability of delirium symptoms by 33.6%. Briefly, worse executive function leads to a significant increased 8-year risk for delirium symptoms.

**FIGURE 2:**
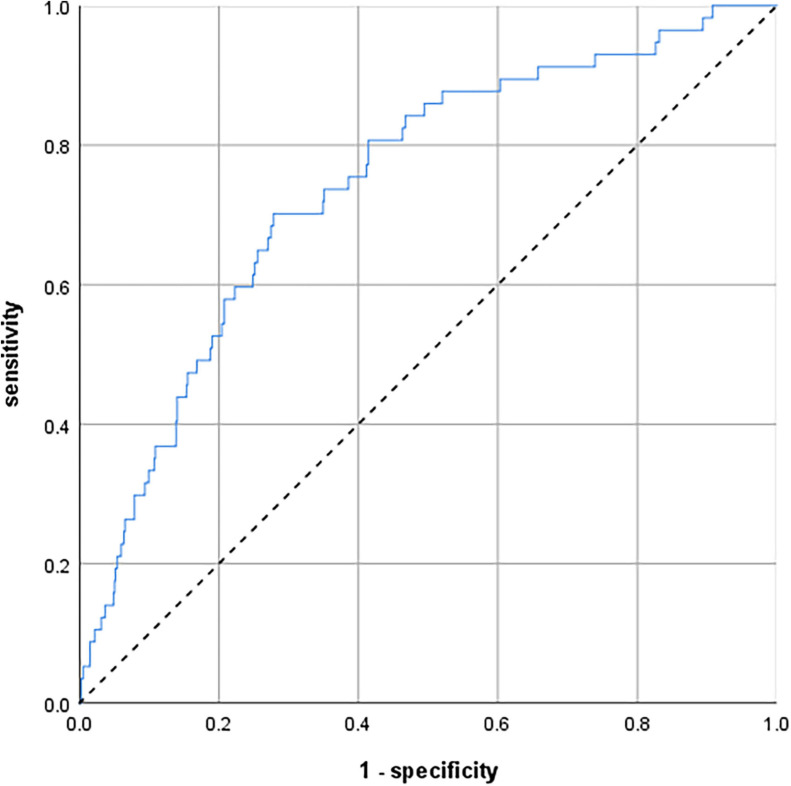
Receiver operating characteristic (ROC) curves illustrating a predicting model to calculate the individual risk of delirium symptoms over 8 years. The model comprises age, comorbidities, depression, and executive function. *N* = 793. Area under the curve (AUC) = 0.743 (95% confidence interval 0.678–0.808).

To predict participants’ individual risk of developing delirium symptoms during the 8-year observation period, we created a model including age, CCI, GDS, and TMT-B. The ROC curve of this predicting model is shown in Figure 2. The AUC was 0.743 (95% confidence interval 0.678–0.808).

### Association Between Impaired Executive Function, Delirium Symptoms, and Dementia

Only 10 participants (1.2%) in our cohort were diagnosed with dementia during the 8-year observation period. Three of them were diagnosed with Alzheimer’s dementia, two each with vascular dementia and mixed-type dementia (vascular/Alzheimer’s). In two patients, the etiology remained unclear. No information was available from 57 participants (7.1%) of our cohort; 42 persons (5.2%) were in ongoing dementia assessment without a confirmed diagnosis of dementia to date. However, the risk of dementia was increased nearly 10-fold for subjects with impaired executive function at baseline assessment (see Table 4). The risk was also increased by a factor of 10 for participants who developed delirium symptoms during the 8-year observation period. If both risk factors are combined, the risk of dementia was even increased by a factor of 48 (odds ratio adjusted for age: 48.759, 95% confidence interval 6.316–376.428, *p* < 0.001).

**TABLE 4:** Multivariate analysis of risk factors associated with occurrence of dementia.

	**Odds ratio**	**95% Confidence interval**	***p-*value**
Age (in years)	1.270	1.107–1.458	**0.001**
Impaired executive function at baseline	10.043	2.274–44.349	**0.002**
Delirium symptoms over 8 years	10.142	2.555–40.251	**0.001**

*Impaired executive function was defined as z score at baseline <-1. Significant p-values are marked in bold. Nagelkerke’s Pseudo-R^2^ = 0.356.*

## Discussion

The aim of this study was to explore the association between deficits in specific cognitive domains and the occurrence of delirium symptoms in an 8-year observation in older non-hospitalized individuals not in need of care. Our data demonstrate that, besides multimorbidity and age, deficits in executive function measured by TMT-B are an independent risk factor for the occurrence of delirium symptoms. Our findings are in line with several previous studies indicating an influence of executive functions on the development of delirium (Rudolph et al., 2006; Greene et al., 2009; Smith et al., 2009). In contrast to those studies that investigated hospitalized patients receiving surgery, our study population consists of non-hospitalized healthy individuals and individuals screened for neurodegenerative risk factors in general. Selection of the cohort took place based on risk factors for neurodegeneration without existing neurodegenerative diseases at the beginning of the study, not for delirium symptoms *per se*. Thus, no selection effects regarding delirium symptoms exist *a priori*. To the best of our knowledge, this is the first study providing the effect of deficits in executive function in non-hospitalized individuals in a large observation period over 8 years.

Authors of most previous studies used short screening tests like the MMSE or MoCA to assess cognitive impairment (Jackson et al., 2004; MacLullich et al., 2009). Whereas short screening tests can be helpful to get a quick insight into subjects’ deficits in general, more extensive test batteries like the CERAD used in this study are more specific and sensitive regarding certain cognitive domains. In our study, we found a significant influence of deficits in executive function on delirium risk. More detailed information about specific domains like TMT-B scores can be helpful in identifying high-risk patients (Greene et al., 2009; Smith et al., 2009). While word list tasks are central in the early detection of dementia, to our surprise, they play a less important role in the prediction of delirium. Accordingly, when selecting a screening, it may be useful to consider tests with included executive function tasks; e.g., the MoCA has some executive function assessment tasks.

In accordance with previous studies (Greene et al., 2009; Smith et al., 2009), we found that depression is another independent risk factor for delirium. Greene et al. (2009) used the GDS and Smith et al. (2009) used the BDI in patients scheduled for surgeries. In contrast, we assessed depression by both the BDI and the GDS in a long-term observational study sample in the older population. Due to the study design, we cannot precisely determine the depressive symptoms at the time of the delirium. However, several authors showed the impact of depression on neuropsychological measures (Snyder, 2013; Brailean et al., 2017), especially on executive functions (Lockwood et al., 2002; Baudic et al., 2004; Patience et al., 2019).

The relationship between depression, cognitive impairment, and delirium has been discussed to be caused by similar pathophysiological mechanisms including disturbances in stress and inflammatory responses (Geda et al., 2006; Panza et al., 2010; Plassman, 2010; Watt et al., 2013; O’Sullivan et al., 2014). On this basis, our clinical findings align with the concepts of inflammaging (Hegeman et al., 2015; Franceschi et al., 2018), multimorbidity, and sickness behavior (Cunningham and MacLullich, 2013), and influence risks and pathological processes of delirium (Wilson et al., 2020). Still, the underlying pathophysiology of delirium has not been completely understood in detail yet (Maldonado, 2018).

Multimorbidity and advancing age are further independent risk factors for delirium symptoms in our study. These results are in line with several previous studies (Fong et al., 2009; Ahmed et al., 2014; Guenther et al., 2016). Considering multimorbidity and age in addition to depression and impaired executive function could be a promising approach to prospectively predict long-term individual delirium risk. In contrast to the studies mentioned above, the number of drugs at enrollment was no independent risk factor for delirium symptoms. This could be for several reasons. Firstly, we only recorded the total number of drugs taken. Drugs that are supposed to increase risk of delirium (e.g., anticholinergic drugs; Pasina et al., 2019) were not recorded separately. Secondly, increasing age and comorbidities often lead to a higher number of prescribed medications. However, in our analysis, we only included the number of drugs at enrollment. Changes in medication in the long-term course over 8 years were not considered. Particularly in depressed participants, it would be interesting to know whether antidepressant medications were taken at the time of delirium. This could reveal interactions between antidepressant treatment and the occurrence of delirium symptoms and would possibly allow conclusions as to which treatment should be encouraged or discontinued before an elective medical intervention in patients at risk. Due to the retrospective study design, we have no information on antidepressant therapy at the time of delirium. Further prospective studies are needed on this issue.

Prevalence of reported delirium symptoms over 8 years was 7.2%. Although there could be an underestimation due to subjects’ amnesia during the episodes of delirium (Oh et al., 2017), prevalence in our study is in line with previous studies investigating older populations without needs for care (Rahkonen et al., 2001; Fong et al., 2009; Witlox et al., 2010; Davis et al., 2012; Krewulak et al., 2018).

In accordance with previous studies (Inouye et al., 2014; Oh et al., 2017), delirium after surgery or invasive interventions (81%) is the leading cause of delirium symptoms in our non-hospitalized study population. Once again, the results underline the importance of perioperative interventions to prevent delirium, especially in older people (Olotu et al., 2019). In our study, we not only considered cases of delirium symptoms unrelated to medical interventions but also included all delirium symptoms, regardless of their trigger factor. Therefore, in contrast to most other studies, we can measure the frequency of delirium symptoms not only in a specific subgroup (e.g., ICU, emergency department, and geriatric wards) but also in a more general older sample.

### Limitations and Strengths

Our study has several limitations: we did not assess acute delirium by standardized and validated screening tools such as CAM or Nu-DESC. However, our novel questionnaire on the history of delirium symptoms is based on the well-established Nu-DESC. Due to our study setup, the use of the well-validated Nu-DESC by assessors was not possible as the delirium was not ongoing at the time points of assessments. Also, reported symptoms could not be validated based on a chart review, as medical notes were not available. Moreover, we primarily asked the subjects themselves and only conducted additional telephone interviews with the relatives or caregivers if the answers in the questionnaire were ambiguous or incomplete. Unfortunately, we did not have a consent to contact the relatives for every subject and, furthermore, some of the participants are living alone. Thus, no standardized third-party anamnesis took place. Shorter deliriums might not be recorded. By basing our questionnaire on the Nu-DESC, there might be an underestimation of hypoactive delirium. Consequently, selective memory about delirium symptoms could be relevant: more recent events were probably to be remembered more likely, vividly, and detailed; also, decline in memory could lead to smaller probability to remember delirium events. Despite this fact, we could rely on the given information as reports were discussed in a multi-professional team and doubtful symptom reports were clarified by talking to subjects and relatives. There were relatively few diagnoses of dementia in our cohort over the 8-year observation period. Even if the number of dementias in our study may be underestimated, this small number already indicates a significant association of impaired executive function and delirium symptoms with the occurrence of dementia.

There are only few studies investigating the risk for delirium symptoms in a broader population-based sample at risk for neurodegenerative diseases with extensive neuropsychological testing over many years in a prospective study (Rockwood et al., 1999; Rahkonen et al., 2001; Davis et al., 2012). Whereas the Vantaa 85 + study (Rahkonen et al., 2001; Davis et al., 2012) and a second cohort study (Rockwood et al., 1999) only used short screenings like the MMSE and CDR, we used the CERAD battery, which allows a differentiated view on certain cognitive subdomains.

## Conclusion

Impaired executive function and depression increase the risk of delirium symptoms over the long term. Screening for these factors can be helpful to identify high-risk patients, particularly before an elective medical procedure, even in relatively healthy subjects (Tieges et al., 2020; Calf et al., 2021). Short neuropsychological assessments like the GDS or TMT-B could be an approach to identify subjects at risk. These tests or shortened adaptations are feasible in hospital routines on surgical wards and thus should be short and easy to use. Using more distinctive measures of cognition, including executive function, patients at risk can be selected for a prophylaxis or prevention program (Zhang et al., 2013) or for a closer surveillance before and after surgery or hospitalization, especially when combined with triggering factors like stress, dehydration, or infection, in accordance with recent guidelines for medical care of delirium (Aldecoa et al., 2017; Hughes et al., 2020). Considering these findings, healthcare costs and individual suffering could be reduced.

### Future Studies

Further prospective studies are necessary to evaluate the interaction of delirium symptoms, executive dysfunction, and depression. As delirium is associated with the risk of a deterioration of health and the development of cognitive decline, a closer look at the described connections can be helpful. Likewise, the used questionnaire should be validated in a larger sample of patients with validated delirium after elective surgery by complete chart review, to control for selective memory or the problem of amnesia for the period of delirium.

## Data Availability Statement

The raw data supporting the conclusions of this article will be made available by the authors, without undue reservation, to any qualified researcher.

## Ethics Statement

The TREND study was reviewed and approved by the Ethical Committee of the medical faculty of the University of Tuebingen (No. 90/2009BO2).

## Author Contributions

CM, US, and KB measured the samples and supported the interpretation of data. MLH, CM, and US interpreted and analyzed the data. CM, MLH, US, KB, A-KT, IW, RY, GWE, and FGM supported the study conceptionally, added to the study protocol, and enrolled patients. US and KB supported the data analysis. CM and MLH contributed to drafting the manuscript. GWE and FGM were the PIs of the study. All listed authors contributed to writing and correcting the manuscript.

## Conflict of Interest

The authors declare that the research was conducted in the absence of any commercial or financial relationships that could be construed as a potential conflict of interest.
