# Supplementary material for: Impaired Executive Function and Depression as Independent Risk Factors for Reported Delirium Symptoms: An Observational Cohort Study Over 8 Years
Source: Front Aging Neurosci. 2021 Jun 7;13:676734. doi: 10.3389/fnagi.2021.676734 (PMC8215445; doi:10.3389/fnagi.2021.676734)
Supplement: Supplementary file 1 [file Data_Sheet_1.docx]

Supplementary Material

# Questions for delirium symptoms (Originally in German)

A delirium or acute confusional state is a temporary disorder that can occur after major medical interventions under anesthesia or sedation (e.g., during a colonoscopy), but also in cases of infection, fever, or disturbances of water and electrolyte metabolism. The following temporary symptoms are typical of delirium:

states of confusion, disorientation, delusions, inappropriate behavior, severe sleep disturbance and memory disorders. These symptoms can occur during one of the above-mentioned events or clinical pictures, but also a few hours to a few days later more frequently in the evening and at night.

1. Have you experienced **any of the following temporary symptoms** **at any time of your life** in connection with a disease/intervention described above?

| **Disorientation** | no | yes |
| --- | --- | --- |
| Temporal disorientation *(you no longer knew what time of day or what date it was)* | □ | □ |
| Local disorientation *(you no longer knew where you were)* | □ | □ |
| Non-recognition of surrounding people *(people who should be familiar to you)* | □ | □ |
| **Delusions or hallucinations** | no | yes |
| Seeing or hearing non-existent things | □ | □ |
| Misperception of things or people *(e.g., coat on wardrobe is misrecognized as a person)* | □ | □ |
| **Inappropriate behavior** | no | yes |
| Removing peripheral venous catheters, infusion tubes or other catheters | □ | □ |
| Incomprehensible or inappropriate communication | □ | □ |
| Incomprehensible aggressive behavior | □ | □ |
| **Sleep disorder** | no | yes |
| Attempts to get out of bed, contrary to given instructions | □ | □ |
| Restraining to the bed was necessary | □ | □ |
| Severe sleep disturbance up to reversed day-night rhythm | □ | □ |
| **Sudden memory/concentration disorder** | no | yes |
| Memory lapses during this time | □ | □ |
| Fluctuation in attention over a period of time (e.g., usual attention in the afternoon, then severe concentration disturbance in the evening) | □ | □ |
| Slowed response on addressing or nudging | □ | □ |
| **Please indicate whether the following is applicable:** | **no** | **yes** |
| Relatives and/or medical staff later reported any of the above behavior/symptoms to you (even if you do not remember them yourself) | □ | □ |
| A doctor has diagnosed "delirium" or "acute confusional state". | □ | □ |

1. Please indicate **when** this/these symptom(s) occurred and **in what context** (e.g., after hip surgery, after a colonoscopy, in the case of a febrile infection):

**Year: ___________________________** (if you do not remember exactly, please estimate)

**In connection with:**

- an operation (e.g., hip operation)
- a procedure performed under sedation (e.g., colonoscopy)
- a febrile infection
- withdrawal of alcohol or sleep-inducing drugs
- other: _______________________
- I had none of the symptoms mentioned above under 1.

1. Please indicate the **temporal relationship between the symptoms and the above-mentioned disease pattern** (if you are not sure, please make an estimate):

- during a specific clinical syndrome (e.g., during infection or alcohol withdrawal)
- immediately after or up to 3 hours after a procedure or clinical syndrome
- 3 hours to 6 days after a procedure or clinical syndrome
- one week or more after a procedure or clinical syndrome
- I did not have any of the symptoms listed above under 1.

1. Please indicate **the duration of the symptoms mentioned above** (if you are not sure, please estimate)

- a few hours
- several hours to several days
- several days to weeks
- a few hours / days spread over several time intervals
- I did not have any of the symptoms listed above under 1.

**If you have answered "yes" to one or more of the above questions (see 1.)**, it would be very helpful for us to be allowed to speak to relatives with whom you have had contact during this period.

- I agree that you may contact the following relative:

Contact details of the relative (name, address, telephone number):

This relative has the following relationship with me: (e.g., wife/ husband, daughter/son)

- I do not consent to relatives being contacted.
- I do not have any relatives.
